# SARS-CoV-2 Molecular Evolution: A Focus on Omicron Variants in Umbria, Italy

**DOI:** 10.3390/microorganisms12071330

**Published:** 2024-06-29

**Authors:** Giulia Bicchieraro, Raffaella Ciurnelli, Alessandro Graziani, Alicia Yoke Wei Wong, Barbara Camilloni, Antonella Mencacci, Roberta Spaccapelo

**Affiliations:** 1 Department of Medicine and Surgery, University of Perugia, 06132 Perugia, Italy; giulia.bicchieraro@dottorandi.unipg.it (G.B.); alessandro.graziani@dottorandi.unipg.it (A.G.); aliciayokewei.wong@unipg.it (A.Y.W.W.); antonella.mencacci@unipg.it (A.M.); 2 Medical Microbiology Section, Santa Maria della Misericordia Hospital, 06132 Perugia, Italy; raffaella.ciurnelli@ospedale.perugia.it

**Keywords:** SARS-CoV-2, Omicron variants, molecular evolution, monitoring, epidemiology, NGS sequencing

## Abstract

Severe acute respiratory syndrome coronavirus 2 (SARS-CoV-2) has caused more than 6 million deaths worldwide, and the spread of new variants over time increased the ability of this virus to cause infection. The Omicron variant was detected for the first time in Umbria, a region of central Italy, in November 2021 and it induced an unprecedented increase in the number of infection cases. Here, we analysed 3300 SARS-CoV-2 positive samples collected in Umbria between April 2022 and December 2023. We traced the molecular evolution of SARS-CoV-2 variants over time through the Next-Generation Sequencing (NGS) approach. We assessed correlation between SARS-CoV-2 infection and patients’ health status. In total, 17.3% of our samples came from patients hospitalised as a consequence of COVID-19 infection even though 81.4% of them received at least three vaccine doses. We identified only Omicron variants, and the BA.5 lineage was detected in the majority of our samples (49.2%). Omicron variants outcompeted each other through the acquisition of mutations especially in Spike glycoprotein that are fingerprints of each variant. Viral antigenic evolution confers higher immunological escape and makes a continuous improvement of vaccine formulation necessary. The continuous update of international genomic databases with sequencing results obtained by emergent pathogens is essential to manage a possible future pandemic.

## 1. Introduction

Severe acute respiratory syndrome coronavirus 2 (SARS-CoV-2), the agent responsible for causing Coronavirus Disease 2019 (COVID-19), has caused millions of infection cases and more than 6 million deaths worldwide from December 2019 to now [1]. From its unclear and debated origin, the virus kept evolving over time, resulting in several variants classified by the World Health Organization (WHO) under the following working definitions: Variant under Monitoring (VUM), Variant of Interest (VOI), and Variant of Concern (VOC). Briefly, a VUM refers to a SARS-CoV-2 variant that has genetic changes that could confer growth advantages but for which epidemiologic impact remains unclear. VOIs, on the other hand, are variants that have genetic changes with known significant community transmission and growth advantage over other variants, while VOCs meet the definition of VOIs and in addition are assessed to cause a detrimental increase in disease severity, impact health systems’ ability to provide adequate care, and significantly impact current vaccine effectiveness [2]. In September 2020, Alpha emerged, the first viral variant classified as VOC, which caused a wave of positive cases, followed by Beta, Gamma, and Delta in 2021. At the end of 2021, a new lineage appeared in South Africa: B.1.1.529. WHO named this lineage as Omicron and designated it as a VOC on 26 November 2021. This variant with its 60 additional selective advantageous mutations compared to the original Wuhan strain rapidly displaced all the other variants present in other countries [3,4,5]. From Alpha to Omicron, a progressive reduction in infection severity and pneumonia cases with an increased tropism for the upper respiratory tract, increased transmissibility, and higher immunological escape was observed [5].

Since the beginning of the pandemic until today, the population-wide immunity evolved, due to both vaccine doses administered and viral diffusion and infections. This resulted in a progressed immune landscape over time, in which viral variants had to move and to evolve to continue spreading and infecting [6].

Molecular biology techniques have proven to be essential for viral detection and variant identification. Next-Generation Sequencing (NGS) and in particular Whole Genome Sequencing (WGS) are the only techniques that allow proper variant identification and classification through the detection of mutations across the genome [7].

In Italy, shortly after the beginning of the virus spread, a network of regional laboratories was established to monitor variants circulating in the population. Italia-COVID19-Genomic (I-Co-Gen) was developed in which reference laboratories upload sequencing data of the SARS-CoV-2 genome [8]. Umbria is part of this network.

In this work, we describe the extraordinary evolution of SARS-CoV-2 variants and its progression in Umbria and compared the results with that obtained in Italy in an early pandemic period, starting from 29 January 2020 when it made its first appearance in Italy with two Chinese tourists in Rome [9]. We focused on SARS-CoV-2 infection in certain categories of people for which the virus is particularly dangerous. Immunocompromised patients, in fact, can result in a prolonged viral replication. Immunocompromised patients have a weak immune response that can result in a prolonged viral replication due to ineffective viral clearance. Moreover, patients that undergo monoclonal antibody therapy can elicit selective pressure on the viral population, resulting in the accumulation of new mutations or recombination events [6,10]. We analysed the SARS-CoV-2 genome with major interest in Spike glycoprotein, which is subdivided into two subunits: S1, which is characterized by two main domains Receptor Binding Domain (RBD) and N-Terminal Domain (NTD), and S2 [10,11]. To date, mutations in the Spike protein have been among the most studied due to (i) the protein’s role in the initial virus–cell contact, (ii) it being the most variable region in the virus genome, (iii) mutations in these sites being demonstrated to enhance viral fitness, and (iv) most importantly increasing immune evasion [11].

The aim of this study was to identify the viral lineages spreading in the population in Umbria, assessing the variant prevalence and consequent COVID-19 infection severity in patients based on age and health status. Data management was carried out using open-source software such as NextClade v2.14.12 [12,13] and databases like the Global Initiative on Sharing All Influenza Data (GISAID EpiCoV) to timely identify and manage the spread of a new virus and share findings [12,14,15].

## 2. Materials and Methods

### 2.1. Sample Collection

This study encompasses respiratory tract samples collected in Umbria, a region of central Italy, from two major hospitals and two local medical centres located in Perugia, Italy (Azienda Ospedaliera di Perugia, and Unità Sanitaria Locale (USL) Umbria 1), and in Terni, Italy (Azienda Ospedaliera di Terni, and USL Umbria 2), during the period of April 2022 to December 2023.

Two sampling strategies for collecting samples to be sequenced were used in this study: continuous flow sequencing and the flash survey. Samples for continuous flow sequencing were taken weekly from specific patient groups, people with severe clinical manifestation, patients that require hospitalization, people admitted to intensive care, reinfections, immunocompromised people, and people returning from countries that are of interest due to the emergence of new variants of public health interest, with the total sample number based on recommendations issued by the Italian National Institute of Health (Istituto Superiore di Sanità (ISS)). The flash survey instead was a monthly random sample collection announced by the ISS where the collection date and number of samples to be randomly collected and sequenced are made known, which is sufficient to provide a “snapshot” of circulating variants at the time of collection [16]. We were able to satisfy the number of samples specified by the ISS for sequencing during the duration of the study period.

As the Azienda Ospedaliera di Perugia is the reference laboratory of the Umbria region, positive samples collected from the four centres were transported for processing there. Respiratory tract samples collected predominantly consisted of nasopharyngeal swabs, and some bronchoalveolar lavage (BAL) samples. Positive samples, defined as those analysed by real-time PCR in the clinical routine of participating medical centres for the presence of SARS-CoV-2 that resulted in having a cycle threshold (Ct) less than 33, were selected for sequencing. All samples with a Ct > 25 and <33 were first concentrated by overnight precipitation at −80 °C with 0.1% ammonium acetate 5 M (Sigma-Aldrich, St. Louis, MO, USA) before proceeding with RNA extraction. Viral RNA extraction was then performed using the Seegene STARlet IVD automated extraction platform together with the extraction kit STARMag 96 X 4 Viral DNA/RNA 200 C Kit (Seegene, Seoul, Republic of Korea) following the manufacturer’s instructions.

### 2.2. Sequencing

For library synthesis, we applied an amplicon-based approach using a COVIDSeq assay kit (Illumina, San Diego, CA, USA), provided by Illumina. First, the reverse transcription of the whole viral genome was performed, followed by the amplification of 98 target regions using different versions of the ARTIC primers’ pool (Artic network [17]) as updated by the manufacturer through the study period based on changes in the SARS-CoV-2 variants circulating. This study used ARTIC primers’ pool V4 from April to May 2022, followed by the V4.1 primers’ pool set from May 2022 to the end of this study that was implemented with 11 additional primers that improve the coverage across amplicons 10, 23, 27, 76, 79, 88, 89, and 90 for the classification of Omicron variants. This resulted in libraries with an average of 300 bp in length. Sequencing of the resulting libraries was performed with the Illumina MiSeq platform by using a Reagent kit (Illumina) with the 2 × 150 paired-end method.

### 2.3. Sequence Analysis

The raw sequence data FASTQ files were generated using the BaseSpace^TM^ FASTQ Generation v1.0.0 app, and the subsequent analysis of the FASTQ files was performed using the pipeline DRAGEN (Dynamics Read Analysis for GENomics) COVID Lineage app v4.0.3 that is freely available in the BaseSpace Sequence Hub Illumina portal. Read alignment to the SARS-CoV-2 reference genome (Wuhan-Hu1/2019) and calling of sequence variants were accomplished using the DRAGEN app. The DRAGEN COVID Lineage app was then used to perform a lineage/clade analysis on this consensus genome using Pangolin [18] and NextClade v2.14.12 [12,13].

### 2.4. Data Sharing and Analysis

Genomic sequences obtained were submitted to the main influenza database GISAID and to the national Integrated Rapid Infectious Disease Analysis-Advanced Research Infrastructure for Experimentation in GenomicS (IRIDA-ARIES) I-Co-GEN provided by ISS [8]. A further analysis of FASTA files obtained by the sequencing of samples collected in Umbria was performed using NextClade v2.14.12 to identify mutations in all viral genomes and to build a phylogenetic tree to analyse variant evolution from the Wuhan-Hu1 strain and lineages’ relationship. GraphPad Prism 8 was used to create a graph representing SARS-CoV-2 variants’ trend in Umbria.

### 2.5. Statistical Analysis

The statistical analysis was performed using GraphPad Prism software version 8.4.3, by two-way ANOVA, with Tukey’s multiple comparison test, to assess the correlation between SARS-CoV-2 infection, patients’ health status, and vaccination doses. Differences were considered significant when *p* < 0.05.

## 3. Results

### 3.1. Demographic Data

From April 2022 to December 2023, a total of 3300 SARS-CoV-2 whole genomes were successfully sequenced, 1798 and 1502, respectively, in 2022 and 2023 (Table 1). Samples were equally distributed according to gender: 47.6% (1571) of samples were collected from males and 52.4% (1729) from females. In total, 73.6% (2430) of patients lived in the province of Perugia while 26.4% (870) lived in the province of Terni (Table 1).

The infected individuals from which SARS-CoV-2-positive samples were obtained were distributed across all age groups: 37.9% were adults over 80 years old (622 in 2022; 628 in 2023), followed by 32.2% aged 61–80 years (537 in 2022; 525 in 2023), 17.2% aged 41–60 (357 in 2022; 210 in 2023), and 8.1% aged 19–40 years (169 in 2022; 99 in 2023), and 4.6% were children under 18 years of age (113 in 2022, 40 in 2023).

Of the 1250 adults over 80 years old, 25 (2.0%) were immunocompromised, 51 (4.1%) were admitted to an Intensive Care Unit (ICU), and 393 (31.4%) had severe clinical manifestation. Of 1062 patients aged 61–80 years, 34 were immunocompromised (3.2%), 39 needed ICU admission (3.7%), and 260 (24.5%) had severe clinical manifestation. Of 567 patients aged 41–60 years, 18 (3.2%) were immunocompromised, 7 (1.2%) were admitted to the ICU, and 59 (10.6%) had severe clinical manifestation. Of 268 patients aged 19–40 years, 2 (0.7%) were immunocompromised and 22 (8.2%) had severe clinical manifestation. Of the 153 individuals of paediatric age, 3 (1.9%) needed ICU admission, and the infection caused severe clinical manifestations in 7 (4.6%) children.

Of the study subjects, 10% were unvaccinated (207 in 2022; 122 in 2023), 0.9% received one vaccine dose (19 in 2022; 11 in 2023), and 7.8% received two doses (154 in 2022; 105 in 2023). The majority (56.0%) of patients received three vaccine doses (1180 in 2022; 670 in 2023) and 17.3% of patients received four doses (177 in 2022; 394 in 2023), while less than 1% received five vaccine doses (15 in 2023). For 246 patients, we did not have any information about the vaccination history (Table 1).

### 3.2. Vaccination Doses in Patients Admitted to the ICU and Immunocompromised Patients

We focused our attention on patients with more serious infections hospitalized in an ICU, or immunocompromised, or those with severe symptoms. A total of 99 infected individuals needed ICU admission, of which 49 were male and 50 were female, with an average age of 77.5 years (ranging from 12 to 99 years of age). Patients that needed ICU admission had a mean interval of 347 days between the last dose of the vaccine and infection, 16.2% of patients had not received any vaccination, 1% of patients received only one dose, 5.1% of patients had received two doses, 52.5% of patients received three doses, and 22.2% of patients received four doses (Table 2). Seventy-eight subjects were immunocompromised, forty-seven were male and thirty-one were female, and had a median age of 70.3 years (ranging from 29 to 92 years of age). Immunocompromised patients had been infected by the SARS-CoV-2 virus with an average interval of 260.8 days from the last vaccine dose, 3.8% had not received any vaccination, 5.1% received two doses, 60.3% received three doses, 20.5% received four doses, and 2.6% had been immunized with five doses (Table 2).

Of 741 patients hospitalized (Table 1), 588 showed severe COVID-19 symptoms, of which 309 were male and 279 were female, with a median age of 77.7 years (ranging from 0 to 100 years of age). Hospitalized subjects had a mean interval of 313.6 days between the last dose of the vaccine and infection, 9.2% had not received any vaccination, 1.2% received one dose, 8.2% two doses, 64.1% three doses, 16.3% four doses, and 0.7% had been immunized with five doses (Table 2).

### 3.3. Prevalence of SARS-CoV-2 in Umbria vs. Italy

**Figure 1:**
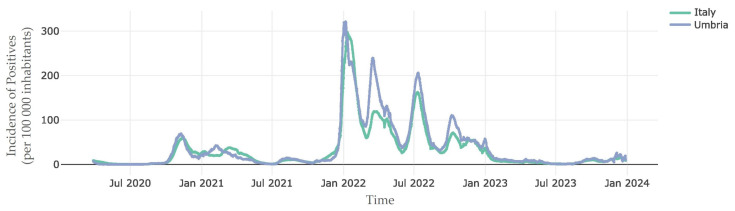
Incidence of positive cases of SARS-CoV-2 infection in Italy and Umbria from the beginning of viral spread in Italy in January 2020 to December 2023 [19].

Italy has been among the countries most impacted by the coronavirus outbreak. We compared the incidence rate of positive cases (100,000 inhabitants) in Italy and in the Umbria region from the beginning of viral diffusion in 2020 to the end of December 2023. After an initial period of low infection incidence, an increase in SARS-CoV-2 infection was observed at the end of 2020, followed by an unprecedented growth in infections especially from the end of January 2022 to January 2023. Regional data were largely similar with the reported national data apart from 2022 where the positive rate was higher in Umbria, and the positive rate waves in our region preceded national incidence peaks (Figure 1).

### 3.4. Phylogenetic Evolution of Omicron Lineages over Time

**Figure 2:**
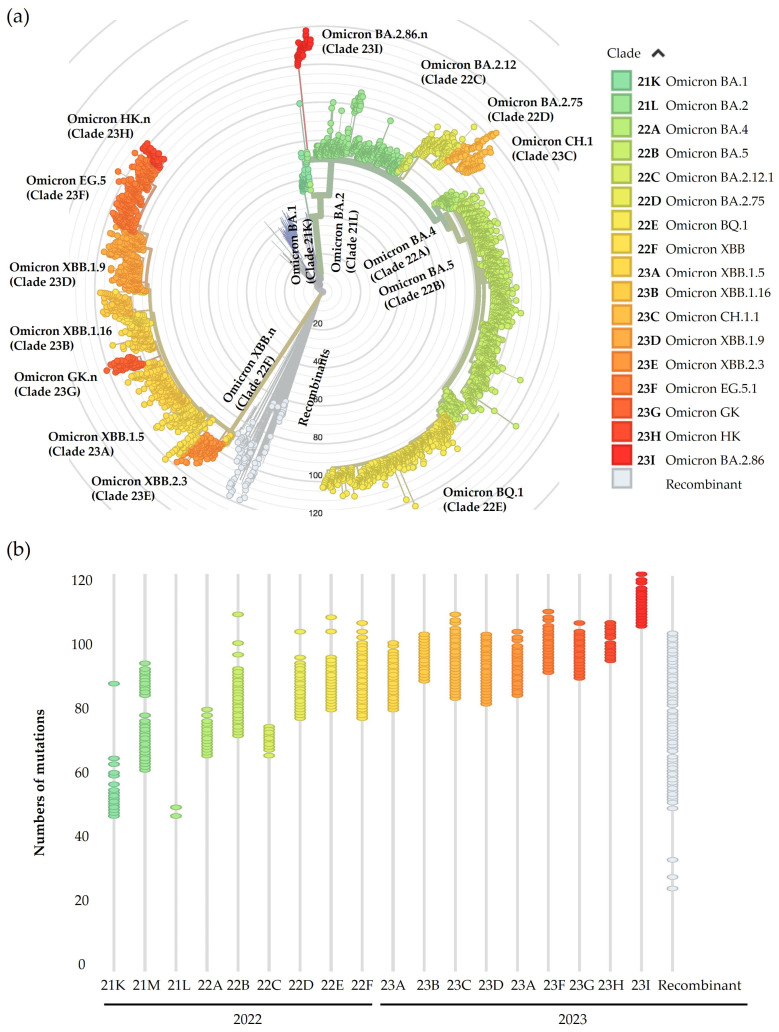
Phylogenetic analysis of samples collected in Umbria from April 2022 to December 2023. (**a**) Radial phylogenetic tree representing lineages identified in Umbria and their divergence from Wuhan-Hu1 strain. (**b**) Nextclade time-tree displaying increased number of mutations in samples based on Nextclade clade classification.

Sequencing results obtained from the 3300 SARS-CoV-2-positive samples were analysed using NextClade v2.14.1 [12,13]. All samples identified during this time period belong to Omicron lineages (B.1.1.529) as follows: 0.7% were classified as BA.1 (22), 20.3% BA.2 (669), 2.0% BA.4 (67), 49.2% BA.5 (1624), and 27.8% were recombinants of the aforementioned lineages (917) [18]. The major Omicron variants identified from April 2022 to December 2023 in Umbria are reported in the phylogenetic tree that represents the viral molecular clock from the original point from which all branches start, corresponding to the ancestor virus SARS-CoV-2 genome isolated in Wuhan City, China (Wuhan-Hu-1/2019, GenBank accession number MN908947.3) [13] (Figure 2a). As the virus gains more mutations with time, the further the distance the virus lineages are plotted from the centre of the phylogenetic tree, thus allowing the tracing of viral evolution. Hence, in the inner part of the graph, there are the parental Omicron variants, from which originate lineages whose nomenclature is reported in the outermost rings (Figure 2a). Figure 2b shows the classification of samples according to their progressive identification, based on NextClade clade names, which consists of a two-digit number representing the year of identification, and a letter that progresses in alphabetical order indicating the order of identification, and the number of acquired mutations that increased over time, starting from Omicron BA.1 with about 65 mutations, followed by Omicron BA.2 with about 70 mutations and the sublineages that depart from the BA.2 branch as BA.2.12, BA.75, and its sublineages as CH.1 (BA.2.75.3), with a growing number of amino acid substitutions, to BA.2.86 with more than 120 mutations. We observed few numbers of Omicron BA.4 variants and a majority of BA.5 from which originates one main sublineage, BQ.1, with about 100 additional mutations in the whole genome. In 2023, almost all variants we identified were recombinant variants. In Figure 2a, recombinant lineages that originated from the recombination between BA.1 and BA.2, and BA.2 and BA.5, are shown in grey, while those that are represented in the shades of yellow and orange all resulted from BA.2.10.1 and BA.2.75 lineages.

### 3.5. SARS-CoV-2 Lineages Detected in Umbria from 2022 to 2023

#### 3.5.1. SARS-CoV-2 Variant Dynamics during the Study Period

**Figure 3:**
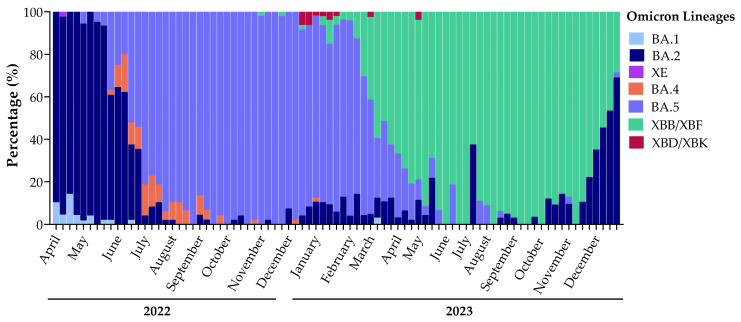
Sequencing analysis results with variants from April 2022 to December 2023 showing the turnover of Omicron variants in Umbria.

The monitoring of SARS-CoV-2 lineages spreading in the population in Umbria through Illumina Next-Generation Sequencing started in April 2022, shortly after the emergence of the B.1.1.529 lineage in viral variants’ landscape (Figure 3). In total, 22 samples were classified as Omicron BA.1 and almost all of them were detected in 2022 (21/22), representing 1.2% of variants identified in 2022 with a prevalence of 8.5% in April, 2% in May, and less than 1% in June. Only one BA.1 variant was identified in 2023 in an immunocompromised patient. The first Omicron BA.2 variant appeared in Umbria in February 2022 [20] and in April, it represented 91.0% of variants detected. We observed a reduction in BA.2 prevalence already in May (86.7%) and in June of the same year, it was overtaken by the BA.5 lineage. BA.2 remained present in the population at a low incidence percentage until the second half of 2023 where the emergence of new BA.2 sublineages, BA.2.75.3.n and BA.2.86, in October was observed. In October 2023, the prevalence of BA.2 sublineages started to increase (7.9%); in November, they represented 10.6% of variants detected and in December, they reached 50.8%. BA.2 made up 27.5% of total variants detected in 2022, which decreased to 11.8% in 2023. The Omicron BA.5 variant was identified for the first time in Umbria in May 2022 and it predominated throughout the year, being positive in 66.9% of sequences. This lineage reached 98.4% of variants identified in October of the same year, when we detected the BQ.1 sublineage (BA.5.3.1.n) that represented the prevalent BA.5 lineage spreading in 2023. In 2023, we observed a radical drop in the BA.5 lineage, which has been superseded by recombinant lineages, but still constituted 28.6% of SARS-CoV-2 variants. Recombinants appeared in April 2022 in Umbria with XE, which is a combination between BA.1 and BA.2 variants. In November 2022, new recombinant variants appeared: XBB, which is the result from the recombination between two BA.2 lineages, followed by XBD, which generated from the recombination between BA.2 and BA.5 lineages. In 2022, recombinant variants represented only 0.8% of the total of sequenced samples, while in 2023, 59.5% of sequences were classified as recombinant (Figure 3).

#### 3.5.2. Evolution of BA.2 Sublineages

**Figure 4:**
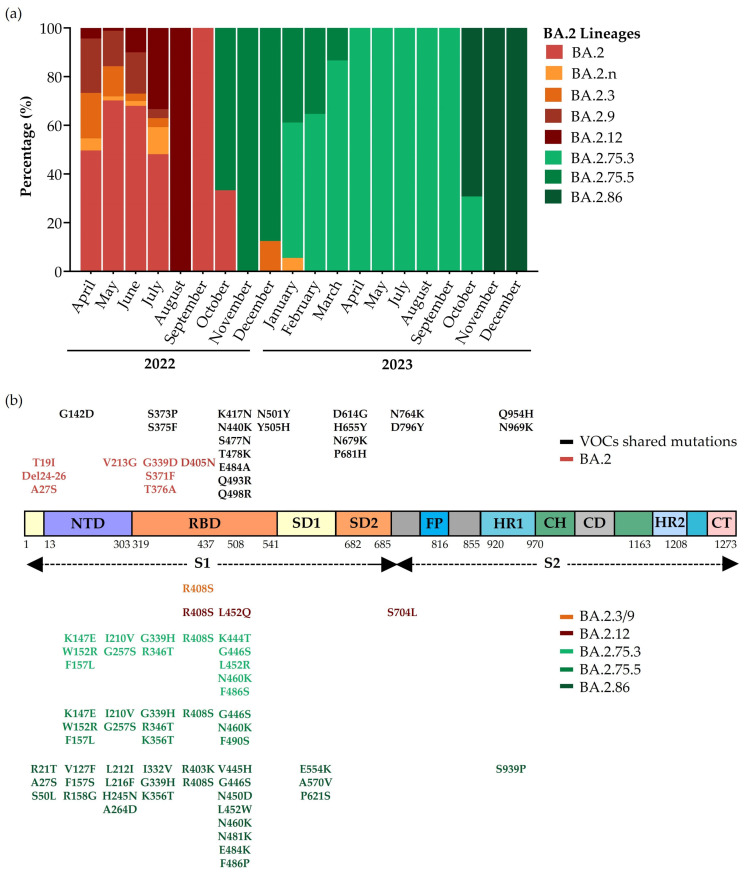
BA.2 lineages’ evolution from 2022 to 2023. (**a**) Turnover of Omicron BA.2 lineages in Umbria. (**b**) Schematic representation of Spike glycoprotein structure and amino acid substitutions of prevalent BA.2 lineages. Abbreviations used: VOC, Variant of Concern; S1, Subunit 1; S2, Subunit 2; NTD, N-Terminal Domain; RBD, Receptor Binding Domain; SD1, Subdomain 1; SD2, Subdomain 2; FP, Fusion Peptide; HR1, Heptad Repeat 1; CH, Central Helix; CD, Connector Domain; HR2, Heptad Repeat 2; CT, Cytoplasmic Tail.

Figure 1 shows three great peaks of a positive rate in Umbria, higher than throughout the rest of the country, between January and July 2022, when Omicron BA.2 appeared and prevailed in the variant landscape (Figure 3). In that period, new sublineages from this variant developed, starting with BA.2.3 and BA.2.9 that share one additional mutation on Spike glycoprotein compared to the parental lineages (Figure 4b). BA.2.3 represented 18.6% of BA.2 lineages in April 2022 but decreased within three months, making its last appearance in December of the same year (12.5% of BA.2 sequences of December). BA.2.9 followed a similar trend and spread in the Umbrian population from April to July 2022 (Figure 4a). In April 2022, we also detected another BA.2 sublineage, BA.2.12.1, with two additional mutations in RBD and one mutation between Spike glycoprotein subunits 1 and 2 (Figure 4b). From July 2022, we observed a significant reduction in BA.2 prevalence (Figure 3), which corresponded to the period in which BA.2.12.1 represented 33.3% of BA.2 lineages circulating in the population, while in August, it reached 100.0% (Figure 4a). At the end of 2022, when BA.2 was no longer prevalent in the population, two new sublineages developed, BA.2.75.3 and BA.2.75.5, with five typical additional mutations in NTD and, respectively, eight and seven additional mutations in the RBD (Figure 4b). BA.2.75.3 lineages represented almost the totality of BA.2 lineages in 2023 and led an increased prevalence over time until the introduction in October 2023 of a new lineage with 57 mutations in Spike glycoprotein: BA.2.86 (69.2% of all BA.2 lineages detected in 2023) (Figure 4b). BA.2.86 induced an increase in BA.2 prevalence especially in December when it represented 50.8% of sequenced samples (Figure 3) when the only BA.2 sublineage was detected in sequences (Figure 4a).

#### 3.5.3. Prevalence of Recombinant Lineages

**Figure 5:**
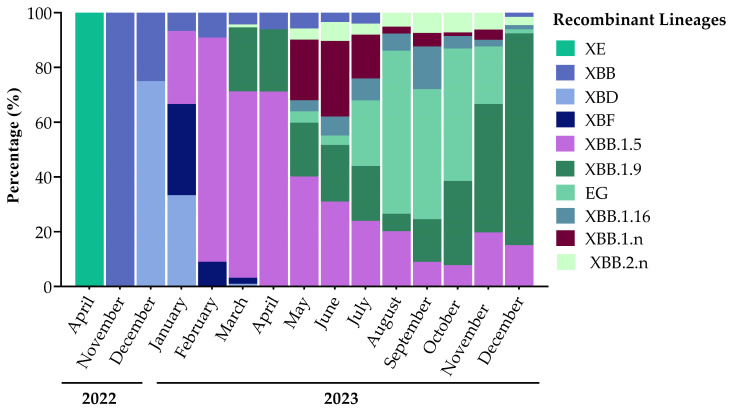
Recombinant variants spread in Umbria from April 2022 to December 2023.

In Umbria, we identified only one sample infected by XE in April 2022. It was the first recombinant that appeared in the viral variant landscape resulting from the recombination between BA.1 and BA.2. The XBB recombinant variant appeared in November of the same year and resulted from a combination between two BA.2 sublineages: BA.2.75.3 and BA.2.10.1 (Figure 5). Recombinants, as is the case for all the other variants, gain mutations over time and thus the sublineage XBB.1.5 developed from the parental lineage XBB, making up 37.5% of detected recombinant lineages. In Umbria, few sequences of XBB.1.5 were detected in January 2023, and in February, the prevalence of this lineage increased up to 81.8% of identified recombinant variants. Its prevalence declined gradually over 2023 but remained present until December (Figure 5). In March, the sublineage XBB.1.9 was detected and differed from XBB.1.5 by one additional mutation in Spike glycoprotein: Q474K in RBD. XBB.1.9 spread in Umbria from its identification to December. From XBB.1.9 evolved the EG lineage, with two other additional mutations in Spike glycoprotein: Q52H and F456L. XBB.1.9 and all its lineages, including EG, represented 50.8% of total recombinants’ sequences. In May 2023, we detected other XBB sublineages: XBB.1.16, characterized by two additional typical mutations in Spike glycoprotein, E180V in NTD and K478R in RBD, and XBB.2.n bearing a new mutation in NTD, D253G. XBB and all its sublineages represented 96.6% of all recombinant variants detected from April 2022 to December 2023 (Figure 5). At the end of 2022, BA.2.75.2 and BA.5.2.1 recombination events resulted in the development of XBD (comprising 1.3% of total identified recombinants), while in January, the combination between BA.2.75.3 and BA.5.2.3 resulted in XBF (0.9% of total identified recombinants). XBD and XBF circulated in the population from December 2022 to March 2023, with always a lower prevalence than XBB lineages (Figure 5).

### 3.6. The Prevalence of Omicron Lineages in Different Patient Groups

**Figure 6:**
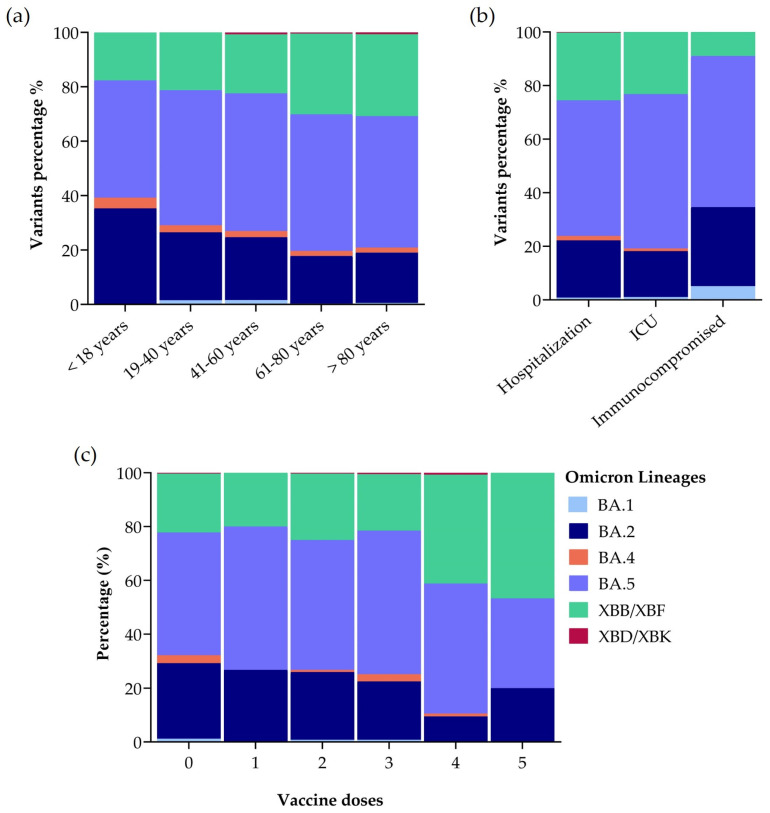
Percentage of Omicron lineages in samples obtained between April 2022 and December 2023. (**a**) Patients were classified based on age groups. (**b**) Patients were classified according to their health status: hospitalized, admitted to Intensive Care Unit (ICU), immunocompromised. (**c**) Patients were classified based on number of vaccination doses. Statistical analysis: Two-way ANOVA, Tukey’s multiple comparisons test.

We ranked sequencing results obtained in the study period according to age groups to observe if there was any significant difference between them. There was no difference between the percentage of Omicron variants infecting different age groups as shown in Figure 6a, but BA.2, BA.5, and XBB were the prevailing variants across all samples. Of patients infected with Omicron BA.2, 35.3% were children aged <18 years, 25.0% were adults aged 19–40 years, 23.1% were adults aged 41–60 years, 17.5% were adults aged 61–80 years, and 18.5% were adults aged over 81 years. Of patients infected by Omicron BA.5, 43.1% were children aged <18 years, 49.6% were aged 19–40 years, 50.6% were adults aged 41–60 years, 50.2% were adults aged 61–80 years, and 48.3% were people over 81 years. Of patients infected with Omicron XBB, 17.6% were children aged <18 years, 21.3% were adults aged 19–40 years, 21.7% were adults aged 41–60 years, 29.7% were adults aged 61–80 years, and 30.2% were adults over 81 years. The prevalence of the XBD/XBK variant was below 1% in adults aged 41–60, 61–80, and >81 years (Figure 6a).

We also stratified sequencing results based on COVID-19 severity and health status of patients: hospitalized, admitted to an ICU, and immunocompromised. Figure 6b shows that also in this case there was no significant difference between the percentage of Omicron variants infecting patients with different health status, but BA.2, BA.5, and XBB were the prevailing variants. Of hospitalized patients, 21.4% were infected with BA.2, 50.6% with BA.5, and 25.3% with XBB, while of the patients that needed ICU admission, 17.2% had a BA.2 infection, 57.6% BA.5, and 23.2% XBB. BA.5 was also prevalent in immunocompromised subjects. In total, 29.5% of immunocompromised patients were infected with BA.2, 56.4% with BA.5, and 9.0% with XBB (Figure 6b). Moreover, we analysed the results according to the number of vaccine doses administered to patients (from 0 to 5) and specific variants present and we did not detect any significant correlation (Figure 6c). BA.5 always represents the predominant variant independently on the number of vaccine doses.

## 4. Discussion

SARS-CoV-2 diffusion and the consequent pandemic provided us an unprecedented opportunity to observe a real-time evolution of a virus and its adaptation to the human host [6]. In this work, we analysed the evolution of the SARS-CoV-2 virus, identifying variants circulating in Umbria from April 2022 to December 2023.

We observed the infection rate in different patient groups, focusing on those that needed ICU entry and immunocompromised and hospitalized patients. We did not observe any gender difference in infected people in our samples. The majority of sequenced samples were classified as random and a flash survey. Of the remaining samples obtained through continuous flow of sampling, the majority (17.3%) came from hospitalized patients due to the viral infection.

The introduction of this antigenically new variant made a vaccine formulation update necessary [21]. Vaccination has been playing an essential role in pandemic evolution, especially the revolutionary application for the first time of mRNA vaccines. The vaccination campaign in Italy began on 27 December 2020 [22]. In our study, most patients received three vaccine doses with a mean interval of about 300 days before the infection, a time frame that is very long for adequate vaccine protection. We also reported that there was no statistic correlation between the number of vaccine doses and specific variants present in the patient population. The European Centre for Disease Prevention and Control (ECDC) and the European Medicines Agency (EMA) stated that an mRNA vaccine booster substantially increased protection, but that protection waned over time; thus, it was recommended to carry out the booster after three months. Evidence showed a high level of protection against severe disease for at least four months after vaccination [23,24].

In Umbria, the positive incidence observed in the region preceded the national peaks of infection, and we speculated that it could be caused by a higher nasopharyngeal swab test rate, especially because of the prevalence of elderly and more fragile populations in Umbria, and its central location in Italy [25].

We officially started the monitoring of SARS-CoV-2 variants circulating in Umbria in April 2022, initially detecting only B.1.1.529 lineages as determined by the Pango Lineage Nomenclature System. As soon as its propagation commenced, different lineages started developing from B.1.1.529. The result was an unprecedented growth in the incidence rate that corresponded to the appearance of Omicron BA.1 first, followed by BA.2 from the end of January 2022, BA.4, and BA.5 in May. Each Omicron variant has characterized additional mutations, like a fingerprint, that allow for unambiguous classification. Carrying all these mutations, Omicron exhibited replication advantages and increased escape ability compared to previous variants [26,27]. Of our samples, 82.6% came from patients that received at least one vaccine dose, thus demonstrating the enhanced antigenic escape of this lineage that also caused an increased risk of reinfection [28]. Variant turnover is clear evidence of viral optimization and adaptation during the progress of the immune landscape [29].

BA.2 seems to be the progenitor of multiple lineages that in some cases develop into other SARS-CoV-2 branches in the phylogenetic tree. The phylogenetic tree shows the evolution of Omicron over time through the addition of an increased number of amino acid substitutions in the Spike glycoprotein. Changes emerged principally in a specific region of Spike glycoprotein: RBD, the ligand that binds the human receptor Angiotensine Converting Enzyme 2 (ACE2) and triggers the conformational change that allows the entry of the virus into host cells. These advantageous mutations had as a consequence a stronger bond with ACE2 and the evasion of humoral immune response [30]. Among BA.2 sublineages, BA.2.75.3 and BA.2.86 prevailed in 2023 in Umbria. From the end of 2022, new recombinant variants started to spread and eventually prevailed over other lineages in the following year. All recombinants, such as XE, XBD, and XBB, originated from BA.2 lineages. XBB was also the origin of other sublineages, in particular EG.5, that overcame the other variants in Umbria and seemed to be the most able to evade immunity through additional mutations gained compared to parental lineages [30,31]. The comparison of our data with that collected in other Italian regions, mentioned in reports of the ISS, highlighted that variants identified in Umbria and their prevalence corresponded to national data, apart from the detection of Omicron BA.3 that was not observed in Umbria [20].

We hypothesized that specific variants could be more prevalent in hospitalized patients or those admitted in an ICU, but our data show that the relative abundance of variants in this target group was in accordance with variants prevalent in all our sequenced samples. With the advent of Omicron, reinfections increased and became a hotspot of study, as described previously [28,32]. We included immunocompromised patients in our investigation. In this group, we observed the prevalence of the BA.5 Omicron variant. Moreover, as observed in previous studies, we identified one immunocompromised patient infected by Omicron BA.1 in 2023, when this variant dropped off the radar from June 2022, that underlines the inability of immunocompromised patients to clear infection [33,34]. Taking this into account, this condition could generate highly mutated SARS-CoV-2 strains with drug-resistant and immune escape properties [35].

In this study, there are some limitations due to an inhomogeneity of sampling. In fact, most patients lived in Perugia, and only 26.4% in Terni. Moreover, 70.1% of sequenced samples came from patients over 61 years old, probably because of a higher testing rate of this age group that is the most affected by the symptomatic and more severe form of infection or because of a population diversity in terms of age distribution in Umbria [25,36]. From the beginning of the pandemic to 2023, restrictive measures changed, and the obligation to test for SARS-CoV-2 lapsed. Therefore, we were not able to quantify neither the number of reinfection cases nor time between infections and variants involved, leading to an underestimation of positive and reinfection rates.

In conclusion, through NGS technology, we identified mutations in the SARS-CoV-2 Spike glycoprotein, which were used to monitor the evolution of variants spreading in the Umbrian population from April 2022 to December 2023. We were able to identify and compare variants infecting different population groups classified by health status and we did not find any significant difference. We likewise did not observe any difference in terms of the infection number between genders, and in terms of viral variants detected in different age groups. Most of our data have been shared and uploaded to open access databases such as GISAID, contributing to variant tracking worldwide. Over time, the impact of SARS-CoV-2 infections on public health evolved and changed restrictions such as mask use. The continuous update of vaccine formulation based on the emerging lineages, as has already been performed for different Omicron variants, and the development of new drugs are connected to variants circulating worldwide. For this reason, SARS-CoV-2 variant surveillance through sequencing analyses is crucial in informing health authorities to come up with appropriate prevention measures and treatment for future emerging pathogens.

## Figures and Tables

**Table 1:** Classification of successfully sequenced SARS-CoV-2 samples obtained from April 2022 to December 2023 by gender, residency, age, reason for genotyping, and vaccination status.

		Total (*n* = 3300)	Patient Samples per Year	
			2022 (*n* = 1798)	2023 (*n* = 1502)
Sex	Male	1571	828	743
	Female	1729	970	759
Province	Perugia	2430	1364	1066
	Terni	870	434	436
Age group	≤18	153	113	40
	19–40	268	169	99
	41–60	567	357	210
	61–80	1062	537	525
	>80	1250	622	628
Reason for genotyping	ICU	99	53	46
	Hospitalization	741	425	316
	Immunocompromised	78	59	19
	Reinfection	115	65	50
	Random choice	2267	1196	1071
Vaccine doses at time of infection	0	329	207	122
	1	30	19	11
	2	259	154	105
	3	1850	1180	670
	4	571	177	394
	5	15	0	15
	Unknown	246	61	185

**Table 2:** Classification of successfully sequenced SARS-CoV-2 samples of ICU patients and immunocompromised and hospitalized patients obtained from April 2022 to December 2023 by gender, vaccine doses, mean age, and days between last dose of vaccine and infection.

		ICU (*n* = 99)	Immunocompromised (*n* = 78)	Hospitalized (Severe COVID-19 Symptoms) (*n* = 588)
Sex	Male	49	47	309
	Female	50	31	279
Vaccine doses at time of infection	0	16	3	54
	1	1	0	7
	2	5	4	48
	3	52	47	377
	4	22	16	96
	5	0	2	4
	Unknown	3	6	0
Mean age (range)		77.5 (12–99)	70.3 (29–92)	77.7 (0–100)
Average number of days between last dose of vaccine and infection (range)		347.0 (52–746)	260.8 (31–729)	313.6 (24–728)

## Data Availability

All virus sequences are available in GISAID (www.gisaid.org).
